# *‘If I donate my organs it’s a gift, if you take them it’s theft’*: a qualitative study of planned donor decisions under opt-out legislation

**DOI:** 10.1186/s12889-019-7774-1

**Published:** 2019-11-06

**Authors:** Jordan Miller, Sinéad Currie, Ronan E. O’Carroll

**Affiliations:** Faculty of Natural Sciences, Division of Psychology, University of Stirling, Stirling, FK9 4LA Scotland

**Keywords:** Organ donation, Opt-out consent, Medical mistrust, Bodily integrity, Family refusal, Qualitative study

## Abstract

**Background:**

There is a worldwide shortage of donor organs for transplantation. To overcome this, several countries have introduced an opt-out donor consent system. This system, soon planned for Scotland and England means individuals are automatically deemed to consent for organ donation unless they register an opt-out decision. This study was designed to explore the reasons underpinning donor choices for people who plan to actively opt-in to the register, take no action and be on the register via deemed consent, opt-out, and those who are unsure of their decision.

**Methods:**

This study reports the analysis of free-text responses obtained from a large survey of intentions towards opt-out legislation in Scotland, England and Northern Ireland (*n* = 1202). Of the *n* = 1202 participants who completed the questionnaire, *n* = 923 provided a free text response explaining their views. Thematic analysis was used to explore the reasons why participants plan to: opt-in (*n* = 646), follow deemed consent (*n* = 205), opt-out (*n* = 32) and those who were not sure (*n* = 40).

**Results:**

A key theme for people planning to opt-in is that it ensures one’s donor choice is *explicitly clear and unequivocal*. Some regarded deemed consent as unclear and open to ambiguity, thus actively opting-in was viewed as a way of protecting against family uncertainty and interference. For the deemed consent group, a key theme is that it represents a *simple effortless choice.* This is important from both a pragmatic time-saving point of view and because it protects ambivalent participants from making a challenging emotive choice about organ donation. Key themes for those planning to opt-out relate to fears around *medical mistrust* and *bodily integrity*. Notably, both participants who plan to opt-out and opt-in perceived presumed consent as “authoritarian” and a method of increasing Government control of organs. In response, registering an active decision protected their freedom of choice.

**Conclusions:**

The findings highlight the importance of registering deliberate active consent for people who choose opt-in, due to concerns over possible family refusal under deemed consent. These findings could inform the development of communication campaigns that encourage family communication before the implementation of opt-out legislation.

## Background

Despite widespread public support for the principle of organ donation around the world, there is a critical shortage of available organs for transplantation. Although transplant activity has increased globally by 7.2% since 2015, there remains an insufficient supply of organs to satisfy demand [1]. To overcome the shortage of organ donors, several countries have changed organ donation laws and introduced an *opt-out* system of consent. This legislative change removes the requirement for individuals to actively sign-up and join the organ donor register (ODR). Rather, an opt-out system follows deemed consent; meaning that if no active donor decision (opt-in or opt-out) has been registered, individuals are automatically deemed to consent for organ donation. If an individual does not want to be an organ donor, they are required to actively opt-out and remove themselves from the ODR. As such, an opt-out system will enable consent for donation to be deemed without the need for people to action an intention to donate [2]. The rationale behind this system is that it should bridge the gap between the publics’ largely favourable intentions towards organ donation and inaction, thus increasing the pool of potential donors. This is important, as although around 90% of the UK public are found to support organ donation, in practice, just 40% have registered as donors [3]. The shortage of UK organ donors is further compounded by poor rates of family consent. Despite continued efforts from the UK Organ Donation Taskforce to increase rates of family consent for donation, they remain low, with 35% of families in Scotland and England refusing consent for donation between 2018/2019 [4]. This is notably heightened for individuals from Black, Asian and minority ethnic backgrounds (BAME), where family refusal rates for donation increase to 58%.

In 2017, the Scottish and English Parliaments announced plans to introduce an opt-out system (also referred to as a deemed consent system). This system has now been in operation in Wales for over 3 years. The latest figures from 2018/19 indicate that the number of donors and subsequent rates of transplantation have now increased, from 181 recorded transplants in 2017/18 to a total of 216 transplants between 2018/19 [5]. Many other countries across the world have implemented an opt-out system of donor consent. The most recent evidence suggests that donation rates (per million population), in countries with opt-out consent systems are, on average higher in comparison to countries with opt-in legislation [6, 7]. However, there is considerable variance found in donation rates, with some opt-in countries having much higher donation rates than opt-out. Moreover, both reviews emphasised that it may be difficult to disentangle the role of other causative factors, including transplantation infrastructure, health care provision, public awareness and underlying public attitudes.

Indeed, a recent review designed to inform the development of the planned opt-out system in Scotland echoed these concerns, reporting limited evidence that in isolation, an opt-out system would increase transplantation rates [8]. The review however, did report strong evidence advocating the importance of public awareness and attitudes towards opt-out consent. This is critical, as extensive research has shown emotional beliefs and attitudes, for example, discomfort at thinking about one’s death, to be key determinants of donor behaviour in countries with opt-in legislation [9–11]. Recent work has also confirmed negative emotional beliefs to be heightened for participants who *plan* to opt-out of the donor register if deemed consent laws are introduced in Scotland and England [12].

### Attitudes towards opt-out consent

Within the last 40 years, there has been a shift in attitudes towards opt-out consent laws in the UK. Evidence from a systematic review of survey data from 1976 and 2007 reported levels of support for opt-out legislation in the UK to vary between 34 and 64% [7]. Notably, the highest levels of support were recorded in surveys conducted after the year 2000. However, the authors note, the review’s conclusions are limited due to methodological inconsistencies in the reporting of the included surveys. As such, the findings may not be fully representative of current viewpoints and attitudes towards organ donation and opt-out consent laws.

While the aforementioned review suggests public support for opt-out laws to have increased, the proposals for opt-out legislation in Scotland and England were met with some controversy. In fact, an independent report from the UK Organ Donation Taskforce advocated against opt-out laws in 2008 over concerns that the system may incur a loss of public faith in the health service and the Government [13]. The belief that opt-out consent increases government control was also echoed within a qualitative report from the Welsh Government prior to the introduction of opt-out consent [14]. Notably, it was these factors that contributed to opt-out legislation being reversed in Brazil [15].

In addition, the introduction of deemed consent may unintentionally result in confusion and ambiguity regarding the role of the family/next-of-kin during the donation decision-making process. Under opt-out laws, it is now the families’ role to confirm whether their loved one had objected to donation, otherwise consent is deemed. Although next of kin will continue to be consulted, in practice, the opt-out system does not provide next-of-kin the legal rights to override or “veto” consent for donation unless explicit evidence of the deceased’s objection is provided [16]. However, international evidence suggests that next-of-kin continue to have a substantial influence during the decision-making process in countries with opt-out consent [17]. For example, after an opt-out system was implemented in Chile, a considerable increase in family refusal rates, and a decrease in donations were reported [18]. The legislation was subsequently revised. Increased rates of family refusal were also reported following the introduction of opt-out laws in Wales [19]. These findings suggested possible confusion among the public regarding the role of the family under the opt-out system [20].

Despite these concerns, little is known about the factors influencing planned donor decisions under opt-out consent laws in Scotland and England. As the Bill for opt-out legislation has now been passed in Scotland and England and is planned for implementation in 2020, a timely investigation of these factors is warranted. Given the emotive nature of the topic of organ donation, the application of qualitative methods may offer a richer understanding of the motivations behind participant’s donor decisions. This study explores the reasons why people plan to make a particular donor choice (opt-in, deemed consent, opt-out and not sure) under an opt-out organ donation system. This data was obtained from free-text responses from a large survey of intentions towards opt-out consent legislation in Scotland, England and Northern Ireland [12].

The aims of this study were: [1] to explore the differences between participants donor choices (opt-in, deemed consent, opt-out and not sure) following the planned introduction of opt-out consent laws, [2] to investigate the key differences between participants who plan to actively opt-in and opt-out of the donor register, and [3] to examine the key differences and similarities between people who provide consent for donation by actively opting-in to the register and those who plan to follow deemed consent.

## Method

### Study procedure

The qualitative data reported in this study was acquired from free-text responses obtained from a questionnaire survey that assessed; previous experience of organ donation, knowledge of organ donation, attitudes towards organ donation and also, examined the donor intentions of participants in Scotland, England and Northern Ireland in a opt-out consent organ donor system. The detailed methodology and results for the quantitative aspects of the questionnaire study are reported elsewhere [12].

**Fig. 1:**
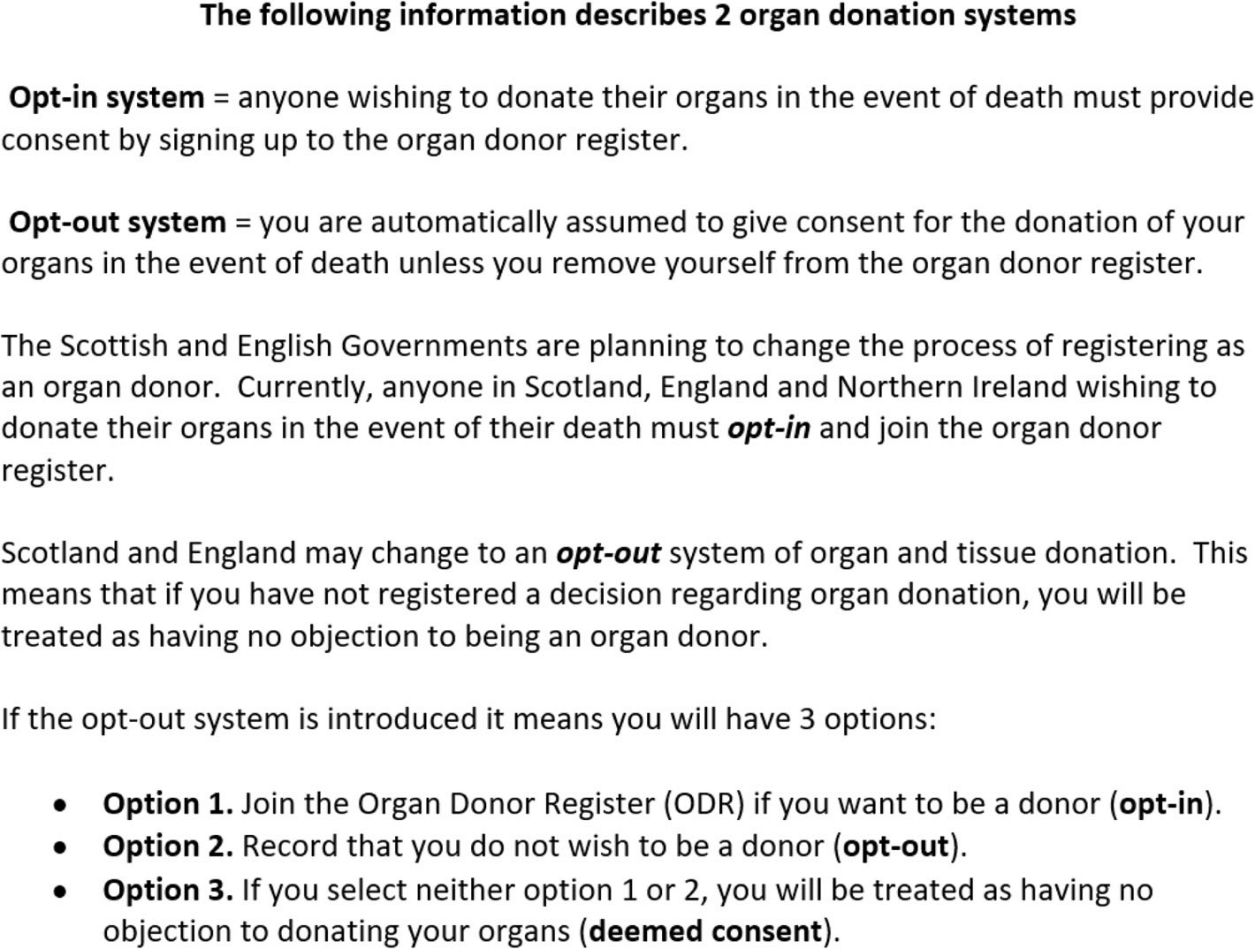
Information presented to participants regarding the planned changes to organ donor laws

The qualitative data in this study describes the reasons underpinning participants donor choice (opt-in, deemed consent, opt-out or not sure) following the introduction of opt-out laws. This was acquired in two stages. To initially obtain a measure of anticipated donor status following the introduction of opt-out consent laws, participants were presented with information describing the proposed opt-out legislative changes (see Fig. 1). Participants were then asked, ‘*If the organ donation laws in your country change to an opt-out system, what would your choice be*?’ The potential responses were as follows; I would opt-in (I want to be an organ donor), I have no objection to donating my organs (deemed consent to be an organ donor), I would opt-out (I do not want to be an organ donor) and not sure. After selecting one of these responses, participants were presented with a free-text entry box and asked to ‘*Please briefly provide the reason behind your choice’.* The qualitative responses obtained from this open-ended response option are the focus of the current study.

### Study recruitment and inclusion

Ethical approval for this study was granted by the General University Ethics Panel at the University of Stirling. Members of the general public over 18 years of age who were currently resident in Scotland, England or Northern Ireland, were opportunistically recruited for this online study. To recruit participants, online advertisements were posted to a university portal page and the social media websites Facebook, Twitter and Reddit. The advertisements presented brief details about the study and contained a URL link to the study, hosted via a Qualtrics questionnaire. Potential participants were initially presented with information about the study before informed consent was requested via an electronic selection box. Aside from three questions addressing consent and eligibility, participants were informed that they could omit a response to any question they felt unable to answer. Recruitment for the study began on the 24th of January and continued until the 12th of March 2018.

### Questionnaire participants

In total, 1202 members of the UK public completed the full online questionnaire. In response to the question: *If the organ donation laws in your country change to an opt-out system, what would your choice be?* 66.1% (*n* = 794) of participants selected to ‘opt-in’ to the donor register, 24.3% (*n* = 292) of participants selected ‘deemed consent to be an organ donor’, 4.2%, (*n* = 50) of participants selected to ‘opt-out’ and 5.2% (*n* = 63) of participants selected ‘not sure’.

### Qualitative free-text responses

Of the total sample of questionnaire respondents, 76.79% (*n* = 923) provided a free-text written response explaining their reasons for this choice. This qualitative data is the focus of the current study. Of these responses, *n* = 646 were obtained from participants who plan to opt-in, *n* = 205 from participants who would follow deemed consent, *n* = 32 from opt-out respondents, and *n* = 40 from participants who selected not sure.

### Data preparation and analysis

The data was analysed using thematic analysis [21]. Thematic analysis was selected due to its flexibility and suitability for identifying patterns within large datasets. The analysis was conducted based on the four respective response groups (opt-in, deemed consent, opt-out and not sure). To enable data familiarisation, each response was read multiple times and preliminary ideas noted. Following this, potential features of interest within each response were systematically highlighted and assigned an appropriate code in MS Word. The responses and respective codes were then organised, reviewed and collated into themes and respective subthemes. To reduce researcher bias, the resulting themes and subthemes were independently reviewed by two members of the research team (JM and SC). Discrepancies in the resulting themes were resolved through discussion with the research team (JM, SC & ROC).

## Results

### Participant characteristics

The age of respondents who provided free-text comments (*n* = 923) ranged from 18 to 82 (*M* = 40.34, *SD* = 12.68). The majority of respondents 80.5% (743) identified as female, 18.3% (169) as male, four participants identified as transgender and seven as “other”. The majority of respondents, 87.3% (806) reported to be living in Scotland, 11.6% (107) in England and 1.1% [10] in Northern Ireland. The demographic information for each response group is presented below in Table 1.

**Table 1:** Demographic characteristics of the opt-in, deemed consent, not sure and opt-out free-text respondents

	Opt-in (*n* = 646)	Deemed consent (*n* = 205)	Not sure (*n* = 40)	Opt-out (*n* = 32)
Age (*SD*)	38.38 (12.04)	43.97 (12.76)	46.47 (12.46)	47.47 (16.22)
Gender				
Male	98 (15.2%)	53 (25.9%)	11 (27.5%)	7 (21.9%)
Female	540 (83.6%)	150 (73.2%)	29 (72.5%)	24 (75%)
Other^a.^	8 (1.2%)	2 (1.0%)	0	1 (3.12%)
Education Level				
Lower Education	277 (42.9%)	90 (43.9%)	20 (50.0%)	16 (50%)
Higher Education^b^	369 (57.1%)	115 (56.1%)	19 (47.5%)	16 (50%)
Employment Status				
Employed	464 (71.8%)	145 (70.7%)	27 (67.5%)	17 (53.1%)
Unemployed	20 (3.1%)	5 (2.4%)	2 (5.0%)	0
Student	94 (14.6%)	20 (9.8%)	2 (5.0%)	4 (12.5%)
Retired	27 (4.2%)	21 (10.2%)	5 (12.5%)	9 (28.1%)
Other	40 (6.2%)	12 (5.9%)	4 (10.0%)	2 (6.3%)
Religious Beliefs^c^				
No Religion	350 (54.2%)	107 (52.2%)	18 (45.0%)	14 (43.8%)
Christian	266 (41.2%)	86 (42.0%)	21 (52.5%)	16 (50.0%)
Roman Catholic	3 (0.5%)	1 (0.5%)	0	0
Jewish	2 (0.3%)	1 (0.5%)	0	0
Other	25 (3.9%)	10 (4.8%)	1 (2.5%)	2 (6.2%)
Organ Donor Status				
Yes	571 (88.4%)	107 (52.2%)	6 (15.0%)	4 (12.5%)
No	35 (5.4%)	72 (35.1%)	31 (77.5%)	28 (87.5%)
Not Sure	40 (6.2%)	26 (12.7%)	3 (7.5%)	0

*Note.*^a^ 4 individuals from the opt-in group identified as transgender. 7 respondents did not state their gender, 4 from the opt-in group, 2 from the deemed consent group and 1 from opt-out respondents. ^b^ Higher education was categorised as completion of a bachelor’s degree. ^c^ Muslim, Hindu and Sikh were included as independent categories however, no respondents reported to follow these beliefs

### Overview of key themes

**Fig. 2:**
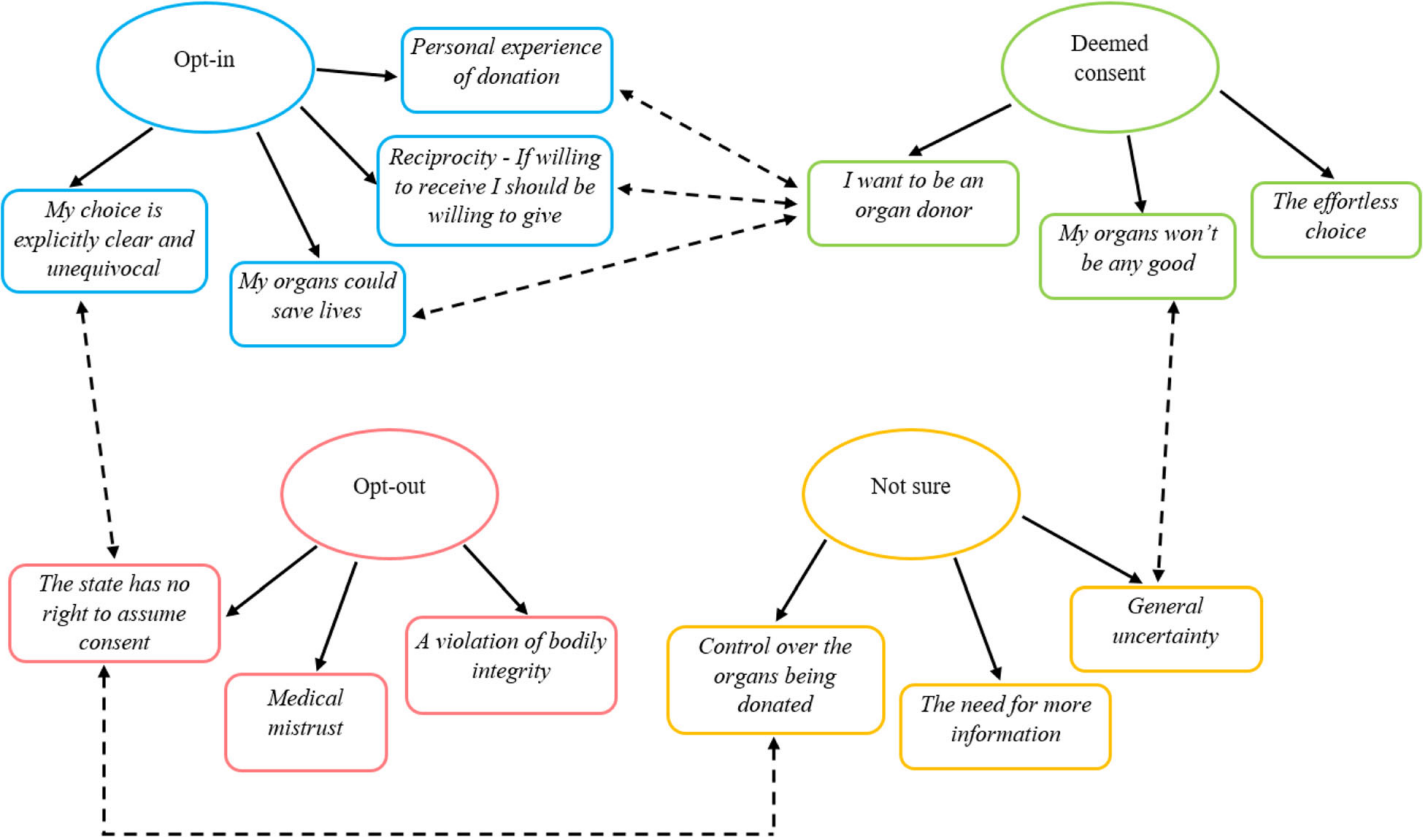
Key themes for the opt-in, deemed consent, opt-out and not sure response groups. Single directional arrows represent the key themes from each donor choice; bi-directional dotted arrows demonstrate a relationship between themes

The overall dataset revealed 13 main themes; organised into each of the four donor response categories (opt-in, deemed consent, not sure and opt-out) see Fig. 2. The four main themes identified for participants who plan to actively opt-in were: (1) my choice is explicitly clear and unequivocal; (2) my organs could save lives, (3) reciprocity - If willing to receive I should be willing to give, and (4) personal experience of donation (please see Table 2 for themes and respective sub-themes). For participants who plan to follow deemed consent, the themes are displayed in Table 3. For participants who are unsure of their decision, the themes are shown in Table 4. Lastly, themes and respective sub-themes for respondents who plan to opt-out of the donor register are available in Table 5.

**Table 2:** Themes and respective sub-themes from the opt-in group

Donor Choice	Themes	Sub-themes
Opt-in	1. My choice is explicitly clear and unequivocal	*- My wishes are set in stone* *- Reduces family distress* *- Protection against family interference*
	2. My organs could save lives	*- Dead people don’t need organs* *- It’s just the “right thing to do”*
	3. Reciprocity - If willing to receive I should be willing to give	(No sub-theme)
	4. Personal experience of donation	(No sub-theme)

**Table 3:** Themes and respective sub-themes from the deemed consent group

Donor Choice	Themes	Sub-themes
Deemed Consent	1. The effortless choice	*- I’m lazy, and this means less hassle* *- It saves me a difficult choice*
	2. My organs won’t be any good	(No sub-theme)
	3. I want to be an organ donor	*- No need for organs when you’re dead* *- Reciprocity* *- Personal experience of donation*

**Table 4:** Themes and respective sub-themes from the not sure group

Donor Choice	Themes	Sub-themes
Not Sure	1. General uncertainty	*- I’m still not sure I want to be a donor* *- Medical uncertainty*
	2. The need for more information	(No sub-theme)
	3. Control over the organs being donated	(No sub-theme)

**Table 5:** Themes and respective sub-themes from the opt-out group

Donor Choice	Themes	Sub-themes
Opt-out	1. Medical mistrust	*- What if I’m not dead?* *- Reduced lifesaving care*
	2. A violation of bodily integrity	*- A desire to remain whole* *- Organ donation damages the body* *- A barrier to a peaceful passing*
	3. The State has no right to assume consent	(No sub-theme)

The illustrative quotes are identified by the corresponding participants’ sex and age, e.g. Female 24 years. Some participants expressed multiple reasons for their choice, therefore some quotes can be assigned to more than one thematic category.

### Themes from opt-in respondents

#### Theme 1: my choice is explicitly clear and unequivocal

This theme represents the view that actively opting-in to the donor register, provides a stronger indication of participants’ wishes to be an organ donor. This theme encompasses three sub-themes, (1) my wishes are set in stone, (2) reduces family distress and, (3) protection against family interference. Throughout, the act of opting-in was reported to signify clear recorded evidence of participants’ donor intentions, in contrast to passively taking no action and following deemed consent. Participants in this group described deemed consent as ambiguous and open to uncertainty. Actively opting-in to the donor register was perceived as a way of ensuring their wishes to donate were explicitly clear and unambiguous (sub-theme 1).

*I wouldn't want any confusion about my intentions to donate my organs after I die so I would opt-in so it was conclusive proof.* (Female 28 years)

*Providing no objection is not actually consenting to donating especially if people are unaware of the system, I think it’s safer to opt-in so it is definitely my choice.* (Female 20 years)

A few participants who plan to actively opt-in perceived opt-out legislation as a threat to their individual responsibility and choice. In the following excerpt, automatically presuming consent for organ donation was thought to indicate the state’s ownership of the body after death. Therefore, the process of actively registering an opt-in decision was viewed as a way of protecting their autonomy and right to decide.

*I do not believe that my body belongs to the state and as such I want to decide what happens after death.* (Male 39 years)

Before organ donation can proceed, the eligible donors’ next of kin are approached and consulted during the donation decision-making process. Participants expressed that by actively opting-in to the donor register and thereby clearly indicating a preference to donate, potential uncertainty and confusion regarding their wishes would be minimised. It was hoped that a recorded informed decision would ameliorate distress when newly bereaved families are confronted with the emotive decision to donate their loved one’s organs (sub-theme 2).

*I would prefer this to be a conscience* [conscious] *decision on my part and not something left to the state. Making arrangements myself would also grant some comfort to my family to know that the harvesting of my organs was something I wanted and not something which was decided for me.* (Male 30 years)

*I would still like to be perceived as an active organ donor, indicating it was my own choice so that my family does not have to make any difficult decision in such difficult time.* (Female 32 years)

Some participants expressed concerns that members of their family, who “don*’*t like the idea*”* of organ donation, may potentially disregard their wishes and overturn their decision. Therefore, for these participants, actively opting-in to the register was a method of safeguarding their choice and preventing potential family interference after their death (sub-theme 3).

*I want there to be no confusion on my death, and I do not want my family (some who do not hold my views) to be upset by, or interfere in my decision after death.* (Female 53 years)

*I want to donate and think the option of “deemed to give consent” is wishy washy and family members could argue against it, saying you didn’t know. I want my choice to be clear.* (Female 41 years)

#### Theme 2: my organs could save lives

Throughout, participants in the opt-in group described a strong desire to save someone’s life as an important reason for opting-in to the donor register. This theme encompasses two distinct subthemes (1) dead people don’t need organs, and (2) it’s just the “right thing to do”. Many participants described simple pragmatic reasons for donating their organs after death and questioned why others did not share the same view. To these participants, their organs were described as personally useless for them, but potentially lifesaving for others (sub-theme 1). As such, the act of discarding functional organs was labelled as “wasteful*”* and “selfish*”*. Often, participants in this response group displayed limited psychological attachment to their organs and likened the act of organ donation to everyday activities such as recycling or donating unwanted items to charity.

*What is the point in letting perfectly good organs rot away inside a dead body when they could be used to save lives? I see it as the same principle as taking things you don't need to the charity shop. Why keep it if you don’t need it but it could really benefit someone else?* (Female 33 years)

*Personally, I just think that it makes sense to donate healthy organs when you can no longer use them yourself. We make a conscious effort to recycle paper etc. so why should it not make sense to recycle valuable organs?* (Female 20 years)

Some participants also expressed the view that ethically, donating their organs after death is simply “the right thing to do” (sub-theme 2). For some, donating organs after death represented their last act of kindness that was somewhat expected as part of their civic and societal duty.

*I believe it would be the most ethical choice, as I would be maximizing my utility to my society through allowing my organs to be given to those who need them most.* (Male 24 years)

*I would like to be able to help someone after my death if I can. Organs are obviously not needed after death it seems like the obvious socially responsible action to take.* (Female 52 years*)*

While, others viewed the act of organ donation as a method of balancing out any negative actions that may have occurred during their lifetime.

*You’re doing a good thing when you're dead. It balances the bad things you've done when alive. A bit.* (Male 44 years)

#### Theme 3: reciprocity - if willing to receive I should be willing to give

Participants who plan to actively opt-in to the register described the notion of reciprocity as an important factor in their decision to donate. Participants explained that if they ever required an organ transplant, a donor organ would be gratefully accepted; therefore, many felt it would be hypocritical not to offer the same opportunity to other people. For some participants, the donor system was likened to a banking system; as such, it was believed that one should only receive what they put in. In this instance, if a person is unwilling to donate their organs, they should not be eligible to receive an organ if they ever needed one.

*I think it should work like a bank if you don't put anything in then you shouldn't be allowed to take anything out. I believe every person who is ABLE to opt in should and those who simply don't fancy it should not be allowed an organ if they find themselves in need of one. If a person cannot donate on genuine medical grounds then they should be allowed to receive an organ if needed.* (Female 39 years)

*If I or one of my children needed an organ I would hope there would be a donor for us, therefore, I expect any of us to be a donor for any other human being in need or an organ. It’s an equalities issue as well as a humanity and compassionate issue.* (Female 47 years)

#### Theme 4: personal experience of donation

Some respondents shared personal experiences of organ donation as the influential factor in their decision to become an organ donor. In the following extracts, participants described how the lives of family and friends had been completely transformed through organ donation. Others described the experience of losing a loved one during the wait for a lifesaving transplant as their motivation for becoming an organ donor. Whilst for some participants, knowing that their loved ones had saved multiple lives by donating their organs upon their death, cemented the importance of organ donation.

*My mum, uncles and papa have all had or having a kidney transplant! My mum had hers 8 years ago and seeing how it's not only changed my life and my dads, but also hers and it’s amazing to see her healthy again!* (Female 23 years)

*My dad needed a liver transplant and I was going to be a “live donor” We had to wait till he was strong enough for the operation but unfortunately he died before we could complete it. Organ donation is giving someone a second chance.* (Male 36 years)

*When my Grandfather passed away, he helped 5 different people from donating organs. This has made me aware of the importance of donating organs and the great difference that it can make for others.* (Female 21 years)

### Themes from deemed consent respondents

#### Theme 1: the effortless choice

This theme encompasses the view that a deemed consent system (taking no action and thereby becoming an organ donor by default) serves as the easiest way of indicating a donor choice. This theme consists of two sub-themes, (1) I’m lazy, and this means less hassle, and (2) it saves me a difficult choice. Participants explained that a system of deemed consent was favourable as becoming an organ donor will now require no conscious effort or unwarranted paperwork to be completed on their part. Many participants expressed a wish to donate their organs yet, had not actively registered as a result of “laziness”. For these participants, deemed consent was viewed as a pragmatic and effortless system that would automatically indicate their wishes (sub-theme 1).

*I'm happy to be opted in without having to fill out any paper work. I hate paperwork so the less I have to do, the better!* (Female 43 years)

*I'm rather lazy and so wouldn't want to have to ‘do’ anything! Besides which I'm happy to donate my organs or whatever's left of them.* (Female 28 years)

Deemed consent was also regarded by some participants as a way of indicating a desire to be an organ donor without the need to make a challenging or emotive decision (sub-theme 2). At times, participants described general support for organ donation yet, reported the thought of organ donation to evoke unease and stated fears of “being cut open after death”. For these participants, deemed consent is considered a way of indicating their underlying preference to donate whilst, avoiding barriers that may have previously prevented these participants from registering an active opt-in decision.

*I’m not against them using my organs after I die but I don’t really want to volunteer for it or anything.* (Female 20 years)

*Opting in means confronting your own mortality, general support for the principal of organ donation and not wishing to confront your own mortality means option 3* [deemed consent] *satisfies both.* (Male 55 years)

*Whilst I don’t want to think about my organs being used or being cut open after death I also think if my organs could be used to save someone that’s acceptable.* (Female 50 years)

#### Theme 2: my organs won’t be any good

Some participants that plan to follow deemed consent, expressed both a desire to donate their organs, however, were unsure if they were medically suitable to be an organ donor due to physical illnesses and poor lifestyle choices. For these participants, taking no action and following deemed consent will enable them to maintain a positive stance regarding organ donation, whilst allowing medical professionals to assess their suitability for donation in the event of their death.

*I would happily opt in but due to a medical condition, I understand they cannot be accepted. I won't actively opt out & leave it to the doctors to decide whether they can use anything.* (Male 57 years)

*Due to being a smoker and overweight I have thought that most of my organs would be of no use. I have no objection to my organs being used if required.* (Female 35 years)

#### Theme 3: I want to be an organ donor

While the aforementioned themes describe the reasons why participants made a choice of deemed consent, the following theme describes participant’s motivations for becoming an organ donor. This is because these participants, satisfied that deemed consent indicated consent to donate, described general reasons *why* they would donate their organs after death. This theme is comprised of three sub-themes: [1] no need for organs when you’re dead, [2] reciprocity, and [3] personal experience of donation. For many, motivations to become an organ donor centred on helping others and giving life after death. Participants frequently stated that after death they would have no requirement for their organs and expressed frustration at the thought of their valuable organs “rotting in a coffin or being incinerated”. Organ donation was subsequently seen as a way of utilising otherwise useless organs to give life to people in desperate need (sub-theme 1).

*I choose it as I have no longer any need for these organs when I'm dead so someone else should benefit from my life - goodness knows I've done bugger all else with it, this is my tiny contribution to humanity.* (Female 42 years)

*Why should we take valuable organs with us when we die, when there are still people who are fighting to live, needing an organ? Organ donation is an amazing thing.* (Female 25 years)

For some, the decision to become an organ donor was driven by the concept of reciprocity. These participants, similar to those in the opt-in group, described feeling duty bound to agree to be an organ donor as they would accept an organ if they or someone close to them ever required a transplant. Therefore, participants felt it would otherwise be “selfish” not to agree to donate (sub-theme 2).

*I'd want an organ if I needed it, so would have to agree with donation.* (Female 39 years)

*I would hope an organ would be available if myself or any friend or family needed. I therefore feel I should be prepared to donate my own organs.* (Female 36 years)

In a similar way to the opt-in group, some participants reflected on their own personal experience of family members whose lives had been transformed through organ donation as their motivation for becoming an organ donor (sub-theme 3).

*My dad waited 3 years for a kidney transplant it transformed his life when he received it. Another friend’s husband had a heart transplant over 20 years ago and he’s still living a full life- it is an honor to help others live full lives once mine ends.* (Female 43 years)

*My father had a heart transplant and would not have survived without the selflessness of organ donors.* (Female 33 years)

### Themes from not sure responses

#### Theme 1: general uncertainty

This theme encapsulates feelings of uncertainty around the decision to become an organ donor and encompasses two sub-themes, (1) I’m still not sure I want to be a donor and, (2) medical uncertainty. Throughout, participants in this group described the enormity of the decision to become an organ donor. Although several participants explained that they had been contemplating organ donation for a long period of time, they remain undecided and uncertain (sub-theme 1).

*I have been thinking about this for many years and am yet to decide what route I'd like to take.* (Female 29 years)

*It’s a big decision and not one that I have ever been confident about making …* (Female 51 years)

*Still really unsure as to whether or not I'm 100% committed to donating organs.* (Male 40 years)

For some participants, pre-existing medical conditions or episodes of physical illness contributed towards uncertainty around the suitability of their organs for use in transplantation. This led to some individuals reporting concerns about transmitting illness’ or poor quality organs to the recipient (sub-theme 2).

*I do not think I am able to donate as I previously had skin cancer. If it would be permitted I would be happy to select opt-out system.* (Female 53 years)

*I have experienced a 2-year period of illness diagnosed as Chronic fatigue syndrome, as I do not know the cause I would not wish others to experience this due to any donation of my organs or blood. Until such times as I can be assured that such would not be the case I would not be happy to donate.* (Male 57 years)

#### Theme 2: the need for more information

Participants felt that they lacked the appropriate information about organ donation in general, and in relation to the proposals for opt-out consent laws. As this legislation has not yet been enacted in Scotland, England and Northern Ireland, many were uninformed of the proposals. For some, more information was required in order to make an informed choice.

*Don’t know enough about organ donation.* (Male 49 years)

*Really don't have enough information, but would like to have more information about this.* (Female 42 years)

#### Theme 3: control over the organs being donated

Participants who were unsure of their decision also described a desire to choose which of their organs would be donated. Some expressed unease at the thought of donating particular organs and tissue, for these participants, a perceived lack of control over the donation process was an important factor in their decision.

*I think not sure covers it. I think there are certain organs I wouldn't want to donate.* (Male 43 years)

*I would like control of which organs are used.* (Female 42 years)

### Opt-out themes

#### Theme 1: medical mistrust

Participants expressed feelings of distrust in the medical profession in the event of life-threatening injuries as a key reason in their decision to opt-out. This theme comprised of two sub-themes, (1) what if I’m not dead?, and (2) reduced lifesaving care. In particular, some participants expressed concerns regarding the validity of a brainstem death diagnosis, and described fears that doctors would hastily harvest their organs before they were really dead. As a result, participants expressed fears that they would be alive and aware of their organs being removed (sub-theme 1).

*I am scared that there would not be enough checks that I was really brain dead before my organs were removed.* (Female 61 years)

*I have no wish to be “kept alive” on a ventilator until my organs are taken out for transplantation on the basis that some doctor has declared me to be “brain dead”.* (Female 65 years)

Participants also voiced concerns regarding the degree of life-saving treatment they would receive if doctors were aware they were organ donors. Throughout participants’ accounts, there appeared to be a dichotomy between donor care and non-donor care. As such, there were concerns that registered donors would receive a reduced lifesaving effort in lieu of saving a potential recipient’s life with viable donor organs (sub-theme 2).

*I have a cynical approach to the care an organ donor would receive in the event of life threatening injuries as oppose to a non-donor in the same position.* (Female 44 years)

#### Theme 2: a violation of bodily integrity

This theme broadly represents concerns expressed by participants that organ donation would violate the physical integrity of their body after death. This theme encompassed three sub-themes, [1] a desire to remain whole after death, [2] organ donation damages the body, and [3] a barrier to a peaceful passing. Participants recurrently expressed concerns that removing organs after death would jeopardise the completeness of their body. It was important for these participants to remain bodily intact after death (sub-theme 1).

*I was born with them I would like to die with them.* (Male 29 years)

*I just want to go out of the world the way I came in.* (Female 47 years)

Participants also reported fears that organ donation would cause unnecessary, additional physical damage to their body after death (sub-theme 2). This was frequently epitomised through powerful word choice that represents harm, e.g. “cut open” when describing the process of organ donation.

*Just do not like the idea of being cut open after death.* (Female 24 years)

*Simply do not wish to be used for any reason after death, put to rest with no damage to body.* (Male 57 years)

Individuals who plan to opt-out viewed organ donation as incongruous to a peaceful passing (sub-theme 3). Throughout, participants expressed distress at the thought of unnecessary medical interventions, such as the use of mechanical ventilation during their death. Participants wanted their death to be a peaceful and natural process; organ donation, however, was believed to delay and interfere with deaths natural course.

*I find it totally gruesome and weird. Let nature take its course and leave things as they are.* (Female 21 years)

*I have no wish to be “kept alive” on a ventilator until my organs are taken out for transplantation on the basis that some doctor has declared me to be “brain dead”.* (Female 65 years)

#### Theme 3: the state has no right to assume consent

Participants held strong views concerning the ownership of their own body. Therefore, this theme encompasses the belief that opt-out consent laws give the government unwarranted control over your body after death. Participants expressed concerns that following the enactment of deemed consent laws, the absence of a clear objection will now be regarded as consent for organ donation. The importance of individual responsibility and informed consent was marked within participants’ responses; this was believed to be threatened under the opt-out system.

*I am a firm believer in individual responsibility and object to the Government making assumptions on my behalf.* (Female 82 years)

*The creation of an opt-out system is inherently wrong in my opinion. The rational conclusion of such a policy is that the state has authority and ownership over your body and organs without ever getting consent.* (Male 22 years)

For some participants, this was considered to criminalise the act of organ donation. In the following excerpt, organ donation under the current opt-in system is viewed as an altruistic gift, yet, under a system which presumes or deems consent, it is theft.

*If I donate my organs it’s a gift. If you take them it’s theft. My body belongs to me. It does not belong to the state to do with as it sees fit. I am a registered organ donor. I will not be if it goes to opt out.* (Female 60 years)

## Discussion

Following the introduction of opt-out consent legislation, if an individual has not registered an active donor choice, consent for organ donation is automatically presumed through deemed consent. This qualitative study prospectively explored the reasons underpinning the planned choice to either, opt-in, follow deemed consent, or opt-out of the donor register following the introduction of opt-out legislation in Scotland, England and Northern Ireland.

### Key similarities between opt-in and deemed consent responses

For participants who want to be an organ donor, either by following deemed consent or by actively opting-in to the register, personal experiences of organ donation were important factors that cemented their decision. Throughout, participants shared emotive anecdotal stories of; loved ones whose lives had been “transformed” after receiving an organ transplant. Others shared experiences of losing a loved one during the wait for a transplant. For many, this personal insight increased their awareness of the importance of organ donation and motivated them to register. These findings are consistent with previous literature, which found personal organ donation experience to be a powerful factor that increases one’s willingness to become an organ donor [22, 23].

Participants in the opt-in and deemed consent group shared a largely pragmatic view of their body after death. These respondents expressed limited psychological attachment to their organs and viewed donation as akin to recycling. Therefore, the prospect of reusing potentially lifesaving organs was an influential factor for both groups.

Another important factor shared by both people who plan to opt-in and follow deemed consent, centred on the concept of reciprocity. Participants in both groups reflected on the impact a donated organ would have if they themselves, or someone they loved required one. This in turn, elicited a sense of obligation to offer the same opportunity to another person. For some, willingly accepting an organ without being willing to register as a donor was considered “*hypocritical”.* The findings from this study support existing research into the use of reciprocity primes and organ donation. For example, priming individuals to think about accepting a donated organ has been found to increase intentions to register as an organ donor [24, 25]. Moreover, research from the Behavioural Insights Team found campaigns that focus on reciprocity to increase active registrations on the ODR. In the aforementioned research, approximately one million participants were exposed to one of eight organ donation campaigns during the process of renewing vehicle tax or registering for a driving licence on the GOV.UK webpage. The campaign that focused on reciprocity by asking, “If you needed an organ transplant, would you have one? If so please help others” was most successful at increasing active donor registrations [26].

Although people who plan to opt-in and follow deemed consent are both indicating a choice to be an organ donor, their reasoning for selecting either an active opt-in or a passive deemed consent decision revealed important distinctions; these are discussed below.

### Key differences between opt-in and deemed consent responses

A predominant reason participants plan to actively opt-in to the ODR when legislation changes, is that it signifies a clear and unambiguous intention to donate organs after death. Conversely, the notion of deemed consent, although largely supported by this group was perceived as being unclear (“*wishy washy*”) and susceptible to ambiguity. Therefore, participants felt that actively opting-in would safeguard their wishes after death. This was important for a number of reasons; for some, this was viewed as a way of preventing family interference, while for others it was hoped that explicitly giving consent would relieve a grieving family of an incredibly difficult choice.

Throughout, participants expressed the belief that the decision to become an organ donor should be a conscious, autonomous choice, and expressed unease at the thought of family members interfering with their decision. To reduce the chance of family interference, participants in this group felt strongly about taking every action possible to ensure their wishes were upheld upon their death. This finding is supported by data from the NHS Blood and Transplant (NHSBT) 2017–18 potential donor audit. This indicated that if an individual had actively registered an opt-in decision, 92% of families consented for organ donation upon their loved one’s death. However, if the individual had not registered a decision to be an organ donor, family consent substantially decreases to 52% [4]. Therefore, in instances where an active donor choice is recorded, consent for donation is markedly higher.

Participants in the opt-in group recognised the enormity and emotive nature of the donation decision-making process faced by grieving families. As a result, many expressed concerns that taking no action and following deemed consent was not sufficiently clear to indicate their wishes to their family. Participants in this group felt that simply providing no objection is not actually consenting, which raised concerns that a grieving family may be left feeling confused. Our findings suggest that opting-in was regarded as an unambiguous way of clarifying donor wishes to families which, would in turn, reduce uncertainty and relieve them of the decision. This is supported by evidence from existing literature, which found confusion and uncertainty regarding the deceased wishes to be a key factor behind family refusal for organ donation [27]. This is important, as data from 2016/17, the year after the introduction of deemed consent laws in Wales, reported 21 instances of family refusal for organ donation. This compared to only eight instances in 2015/16 [19]. The heightened rates of family refusal were attributed to uncertainty regarding the role of the family under the opt-out system [20]. As a result, an extensive communication campaign that focused on encouraging people to share their donor wishes with family and friends was introduced. Family consent rates in Wales are now 70% and highest in the UK [4]. Therefore, before the introduction of deemed consent laws in Scotland and England, the development of campaigns that encourage families to discuss their wishes should be a priority.

The enormity and emotive nature of a donor decision was also a recurrent theme expressed by participants who plan to follow deemed consent. However, for some participants in this group, taking no action and thereby becoming an organ donor by default was viewed as a way of avoiding this difficult choice. The findings of this study suggest participants experienced ambivalence when considering organ donation; simultaneously describing both support for organ donation whilst citing fears and emotional barriers as a major deterrent to a donor choice. The concept of ambivalence and affect is recognised as an important factor for donor relevant decisions [28]. The participants in the present study described fears of being “*cut open after death*” or general discomfort about “*confronting your own mortality*” when contemplating a donor relevant decision. These factors are recognised deterrents to donor registrations [9–11]. However, this was also accompanied by affirmations of support for the principle of organ donation. Therefore, deemed consent emerged as a preferred option by some, as it signifies the underlying wish to be an organ donor without the need to confront aversive emotional barriers during the active registration process. Ultimately, this system may increase the pool of potential donors by including ambivalent individuals who previously felt unable to register an active opt-in decision yet want to donate their organs.

The effortless nature of deemed consent may also increase the pool of donors; by including people with favorable viewpoints towards organ donation who have not registered an active opt-in decision as a result of “*laziness*”. Throughout, participants in this group favored the simplistic nature of a deemed consent system as, unlike the current opt-in system, consent can be recorded without any required action. Consistently, research has shown that one’s positive intentions do not exclusively predict behavior [29]. Organ donation is a particularly powerful example of this tendency; as although the majority of the UK public support organ donation, just 40% of people are registered as donors [3]. Therefore, a default system that removes the requirement for active registration may reduce this discrepancy by capturing those who have not yet actioned their intentions.

### Key differences between opt-in and opt-out responses

For the participants who plan to actively opt-out of the donor register, fears surrounding the medical profession were salient among participants’ reasons for opting-out. Notably, this concerned the validity of using brainstem death criteria as a method of defining irreversible total-body death. The complex and misunderstood nature of brainstem death has been recognised in previous qualitative research in organ donation [30]. Participants who plan to opt-out often did not equate brainstem death as a “real” death; this manifested as fears of premature withdrawal of care and donation occurring while patients were still alive. To ensure potential donor organs are in optimal condition, they require an adequate supply of oxygen. As the patient’s breathing is maintained using mechanical support, it can be challenging for families to comprehend that the person is no longer alive. These views may also be compounded by misleading depictions of brainstem death and organ donation portrayed within the media [31]. As the main source of information and knowledge regarding organ donation, damaging media misrepresentations rapidly propagate and influence the development of harmful beliefs [32].

Concerns that organ donation would violate the physical integrity of the body were also prominent within participants’ reasons for planning to actively opt-out. Expressions of bodily integrity concerns were expressed through fears over a loss of completeness without organs, disfigurement and concerns over unnecessary intervention to prevent a peaceful death. Participants expressed worries that organ donation would involve “*cutting-up”* the body for organs to be “*harvested”.* Such concerns have consistently emerged as key factors that deter potential registrants [9–11]. The concept of bodily integrity is rooted in mortality and personal autonomy [33]. As these beliefs centre around transgressions of the body after death and have consequences for the afterlife, they are intrinsically challenging to falsify and overcome. These may be exacerbated by conflicting viewpoints of organ donation and religion. Although major religions in the UK support organ donation, a recent UK survey found that over 50% of respondents believed organ donation was against most religious beliefs [12].

Another reason for choosing to opt-out focused on perceptions of heightened government control of organs after death. The importance of free choice and autonomy were central reasons for opting-out; signifying that participants’ choice was perceived to be threatened under proposals for opt-out. This may reflect the wider psychological concept of reactance, an unpleasant emotional response experienced following a perceived threat to one’s freedom [34]. Consequently, perceptions of presumed consent as an impingement of rights may result in the public taking action to protect their free choice (*opting-out*). Indeed, some critics of opt-out legislation have reported that accepting the absence of objection as permission for donation, to undermine the ethical principles of informed consent [35]. Moreover, the absence of active informed consent was perceived as reducing the altruistic nature of organ donation to an act synonymous with theft. Although concerns over government control were reported in Wales prior to the introduction of opt-out consent laws, limited research has investigated these issues. Given such concerns have contributed to the reversal of opt-out consent in other countries [15] a timely exploration of these factors is necessary.

The concept of reactance was not exclusively reported by individuals who plan to opt-out but was also evident among individuals who plan to opt-in. Interestingly, although this group plan to actively register as organ donors, some perceived the plans to introduce deemed consent as a coercive action from the government. Our findings suggest that a sense of ownership over one’s body is important for both those who plan to opt-in and opt-out. Therefore, registering an active donor choice was viewed as a way of safeguarding particpants’ freedom of choice and eliciting control over their donor decision.

#### Implications for future research

This study has important implications that may inform future research and practice. In particular, our findings demonstrate the importance of encouraging clear unambiguous consent for people who would opt-in, and illustrate concerns of confusion and the potential for family refusal under deemed consent. This finding could inform the development of communication campaigns in Scotland and England that focus on explaining the role of the family before the implementation of opt-out laws. This is important, as after the enactment of opt-out legislation in Wales, instances of family refusal doubled [19]. Now, following extensive campaigns designed to encourage family communication and to prevent families from overturning their loved one’s wishes, Wales has the highest rate of family consent for organ donation in the UK [4]. This is particularly important for individuals within BAME communicates, where family refusal rates are markedly higher. Given that 21% of individuals who died while waiting for a transplant last year were from BAME communities, there is an urgent need to increase family consent and rates of donation among minority ethnic groups [36]. Secondly, our findings also highlighted fears of medical mistrust and perceptions of government control under opt-out laws. To reduce the number of people planning to opt-out of the organ donor register, the evaluation and development of targeted campaigns to challenge concerns of medical mistrust and heightened government control under opt-out laws are urgently required.

### Strengths and limitations

It is important to first acknowledge some limitations of our study. As is common in this field, there was a recruitment bias, in that the majority of free-text responses were obtained from female participants and individuals living in Scotland. We also recognise that there were a limited number of participants from BAME communities. Secondly, as survey methods do not permit exploration or probing of salient response topics, the use of a questionnaire will have to some degree limited the depth of participants’ responses. However, the use of an open-ended free-text response option, enabled participants to explain in their own words, the reasons important to them. As such, the data was suitably rich and detailed to provide a breadth of information and insights into donor decisions under opt-out consent. Although interview-based methods are preferred in qualitative research, the number of studies collecting data using questionnaire-based methods are increasing, particularly when exploring potentially sensitive topics [37, 38]. This method also has a number of strengths. Importantly, the use of an online survey was effective at obtaining a large sample of over 900 respondents across different donor choices (opt-in, opt-out and deemed consent). This is, to the authors’ knowledge, the largest qualitative study to examine donor decisions under the new opt-out organ donation system. A particular strength of this method is that it may reduce socially desirable responding often experienced when using quantitative methods to investigate potentially emotive topics, such as in this study [39]. Moreover, these methods offer participants anonymity to express potentially complex and contentious viewpoints. This is particularly important for people who plan to opt-out of the donor register who may be hesitant to express their decision not to donate within a face to face or group setting. Before the introduction of opt-out laws in Scotland and England, future research using qualitative interview methodology is warranted to obtain a comprehensive understanding of the factors influencing donor decisions under opt-out consent.

## Conclusion

This research provides deeper insights into donor relevant decisions following the enactment of opt-out consent legislation in Scotland and England. The findings highlight the importance of an active indisputable choice for individuals in the opt-in group, to ensure their wishes are safeguarded and not overridden by distressed families at the time of death. The introduction of deemed consent is advantageous primarily for those who have not actioned intentions to be an organ donor due to “laziness”, and for those with psychological ambivalence towards organ donation as it protects them from making a difficult choice. Our findings from participants who plan to opt-out, reinforce the existing opt-in organ donation literature around concerns of medical mistrust and violations of bodily integrity, and highlight a novel deterrent for the opt-out system, namely concerns of heightened government control. Although implementing a system of deemed consent may increase the pool of eligible organ donors, the potential for confusion should not be overlooked. Two primary concerns with deemed consent, family interference and reactance due to perceptions of unwarranted government control have emerged from this study. Before the introduction of deemed consent laws, the development of campaigns to target these factors is imperative.

## Data Availability

The datasets used during the current study are available from the corresponding author upon reasonable request.

## Abbreviations

BAME: Black, Asian and Minority Ethnic
NHSBT: NHS Blood and Transplant
ODR: Organ Donation Register
UK: United Kingdom
